# Palmitoyl-glucosamine co-micronized with curcumin for maintenance of meloxicam-induced pain relief in dogs with osteoarthritis pain

**DOI:** 10.1186/s12917-023-03594-4

**Published:** 2023-02-07

**Authors:** Giorgia della Rocca, Carlo Schievano, Alessandra Di Salvo, Maria Beatrice Conti, Maria Federica della Valle

**Affiliations:** 1 Department of Veterinary Medicine, Centro Di Ricerca Sul Dolore Animale (CeRiDA), Università Degli Studi Di Perugia, 06123 Perugia, Italy; 2 Innovative Statistical Research SRL, Prato Della Valle 24, 35123 Padua, Italy; 3 CeDIS (Centro di Documentazione e Informazione Scientifica), Innovet Italia SRL, Via Leonardo da Vinci 3, 35030 Saccolongo, Italy

**Keywords:** Osteoarthritis, Dogs, Chronic pain, ALIAmide, Palmitoyl-glucosamine, Curcumin, Meloxicam, Non-steroidal anti-inflammatory drugs, Multimodal analgesia

## Abstract

**Background:**

Osteoarthritis (OA) pain is the number one cause of chronic pain in dogs. Multimodal treatment, including combining safe and effective nutritional interventions with non-steroidal anti-inflammatory drugs (NSAIDs), is currently considered one of the most appropriate choices for managing OA pain. Palmitoyl-glucosamine is a feed material belonging to the ALIAmide family, whose parent molecule is the prohomeostatic lipid amide N-palmitoyl-ethanolamine. Curcumin is a promising plant antioxidant. The present study aimed at investigating whether 18-week dietary integration with palmitoyl-glucosamine co-micronized with curcumin was able to maintain pain relief in dogs with OA-associated chronic pain receiving meloxicam (1.5 mg/ml oral suspension) on a tapering regimen (progressive 25% decrease of the original 0.1 mg/kg/day dose, on a biweekly basis) during the first 8 weeks of treatment. Pain was assessed both by the owners and veterinary surgeons, with the first using both subjective evaluation and validated metrology instruments—i.e., Helsinki Chronic Pain Index (HCPI) and Canine Brief Pain Inventory (CBPI)—while the second rating the severity of lameness and pain on palpation on two previously used 5-point scales.

**Results:**

A total of fifty-eight dogs with OA chronic pain entered the uncontrolled study. Pain on HCPI was considered severe at baseline (range 18–39). Based on owner’s assessment, 90% of dogs who responded to meloxicam at the full-dose regimen could reduce meloxicam up to 25% of the original dose without experiencing pain worsening. Moreover, 75% of dogs was assessed as having no pain increase ten weeks after meloxicam withdrawal. A statistically significant decrease of pain severity as scored by HCPI (*P* < 0.0001) was observed two and ten weeks after meloxicam withdrawal compared to study entry (17.0 ± 1.05 and 15.1 ± 1.02, respectively, *vs* 29.0 ± 0.74; mean ± SEM). After meloxicam withdrawal, no statistically significant change in the CBPI scores was recorded. Pain on palpation and lameness significantly changed to less severe distributions along the study period (*P* < 0.0001).

**Conclusion:**

The findings appear to suggest that dietary integration with palmitoyl-glucosamine co-micronized with curcumin was able to maintain meloxicam-induced pain relief in dogs with severe OA chronic pain.

## Background

Osteoarthritis (OA) is a chronic degenerative disease of the whole joint and one of the leading cause of persistent and chronic pain in dogs [1, 2]. OA pain may result from the activation of local nociceptors by inflammatory substances released by immune cells, like synovial macrophages and mast cells [3]. It may also originate from muscle spasms, subchondral bone microfractures, mechanical stretching of joint nerve fibers due to excessive distension of the joint capsule and impinging of joint structures by osteophytes [4]. In the event nerve fibers get involved and damaged, neuropathic pain may arise [5, 6].

Importantly, inflammatory and neuropathic pain are both responsible for morpho-functional and behavioral as well as lifestyle changes, like muscle atrophy secondary to disuse, aggressive or submissive behavior, altered sleep–wake rhythm and impaired social interactions [7].

Given the dog’s inability to self-report, pain recognition and assessment will necessarily require particular tools different from self-report instruments commonly used in humans. It is therefore imperative to recognize, measure and monitor OA pain in dogs with validated metrology instruments, i.e., questionnaire-based measurement tools, which assess changes in pain-related behaviors in the dog’s home environment and have been developed to quantify pain and assess outcome [8, 9].

Moreover, OA pain must be viewed as a multifactorial problem and a multimodal approach should be considered, concurrently addressing degenerative and inflammatory pain pathways within the joint. This consists in the combined use of pharmacological and non-pharmacological interventions targeting different steps of OA, in order to achieve the best pain relief and reduce drug use, either in terms of dosage and treatment duration [10]. In this scenario, combining nutritional interventions with classically used drugs, like non-steroidal anti-inflammatory drugs (NSAIDs), is considered an effective strategy [10, 11]. Indeed, although NSAIDs are commonly and successfully used in OA patients [12–17], their long-term administration has potential disadvantages mainly in terms of increased incidence of adverse events [18]. NSAID dose reduction has been suggested as a possible solution [19].

Among the dietary interventions for OA and related pain, chondroitin sulfate and glucosamine have long been used in veterinary medicine and proved to slow OA progression [20], although they appear to have no effect on OA pain [21, 22]. Omega-3 fatty acids have been shown to reduce some clinical signs of canine OA, with findings relying on subjective assessment only [23, 24]. A substantial need remains for dietary interventions that are safe and effective in targeting OA pain on the basis of objective evaluations. Some evidence is currently accumulating on a novel dietary supplement for OA pain, i.e., N-palmitoyl-D-glucosamine (PGA), the amide of palmitic acid and glucosamine. PGA belongs to the ALIAmide family (Autacoid Local Injury Antagonist amides), whose parent molecule is the naturally occurring and prohomeostatic compound N-palmitoyl-ethanolamine (PEA) [25]. Like PEA, PGA, is a highly lipophilic compound (predicted log *P* value of 5.6). Once the bioavailability limitations due to its high lipophilicity are overcome through particle size reduction (i.e., micronization) [26], PGA has shown protective effects in preclinical models of inflammation and chronic pain [26, 27]. Multiple cellular and molecular mechanisms sustaining PGA effects have been suggested, among which (i) the down modulation of synovial mast cell degranulation and hyperplasia, (ii) decrease of pro-inflammatory cytokines and growth factors, (iii) intracellular release of glucosamine through enzymatic cleavage, and (iv) toll-like receptor 4 (TLR4) antagonism [26, 27]. Interestingly, PGA is listed in the EU‐Catalogue of feed materials used in animal nutrition.

Accumulating evidence shows that the antioxidant plant polyphenol curcumin may offer a complementary anti-inflammatory support for OA dogs [28–30]. A co-micronized formulation of PGA together with curcumin (PGA-cur) has been developed and preliminarily investigated in murine models of inflammation and OA pain. In particular, dietary supplementation with PGA-cur significantly reduced clinical and histopathological signs of inflammation and pain in carrageenan-induced edema and hypersensitivity in the rat [31]. Moreover, it protected articular cartilage against degeneration and counteracted OA-associated increase of proinflammatory cytokines as well as sensitizing mediators (e.g., nerve growth factor) and matrix metalloproteases in a rat monoiodoacetic acid-induced model of OA pain [31]. Finally, PGA-cur reverted OA allodynia and locomotor dysfunction in the same model [31]. The latter effects have also been confirmed in a preliminary survey on 181 privately-owned OA dogs being managed with conservative treatment along with the add-on use of PGA-Cur [32].

Therefore, PGA-cur may well represent a promising tool in the multimodal management of OA pain, given its ability to counteract nerve sensitization and degradative as well as inflammatory pathways within the joint.

The hypothesis of the present study was that dietary supplementation with PGA-cur administered concurrently and consecutively to a reference NSAID (i.e., meloxicam) was able to maintain the latter’s full-dose effect in dogs with OA associated chronic pain, despite the progressive dose reduction or discontinuation.

## Results

### Flow chart and dropouts

**Fig. 1:**
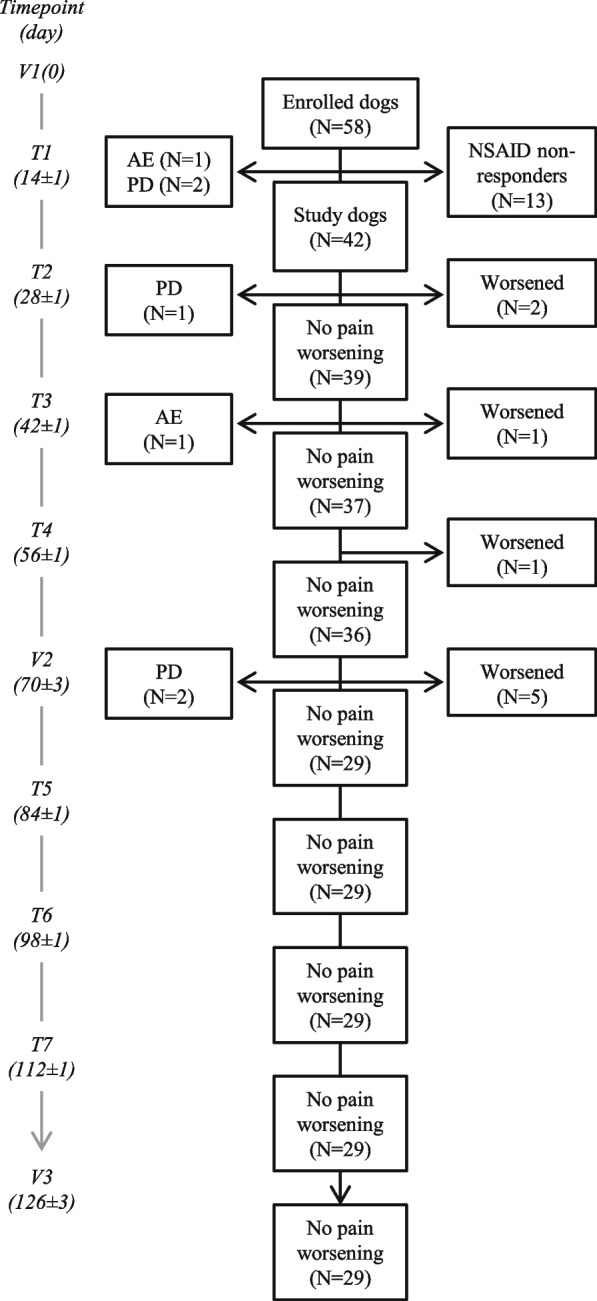
Flow chart of the study. The flow chart indicates the sample size of enrolled dogs and withdrawals due to (i) failure to respond to the NSAID, (ii) adverse event and (iii) protocol deviation. Time and number of dogs experiencing pain worsening is also indicated. On the left-hand side a schematic timeline is given for convenience (see "Methods" for further details). Abbreviations: AE, adverse event; PD, protocol deviation; T1-T7, first to seventh telephone interview; V1, V2, V3, first (i.e., baseline), intermediate and third (i.e., final) clinical visit, respectively

Fifty-eight dogs were included by nineteen privately owned veterinary clinics. The flow chart of the study is depicted in Fig. 1.

Thirteen dogs (22.4%) did not succeed in reaching at least 30% pain reduction at HCPI during the first 2 week-treatment (i.e., NSAID at full dose) and were considered “NSAID nonresponders”.

Besides NSAID nonresponders, three dogs dropped out during the first 14 days for reasons unrelated to lack of pain reduction, with one experiencing an adverse event and the others due to protocol deviations (no-show at T1). Thus, 42 dogs proceeded with the study and are referred to as “study dogs”.

In the following weeks, one more dog dropped-out due to an adverse event, three dogs exited the study because they needed surgical intervention unrelated to the orthopedic condition, and nine dogs experienced pain worsening according to their owner’s assessment (Fig. 1).

In accordance with the aim of the study, NSAID nonresponders were excluded from the outcome analysis. Nonetheless, their baseline characteristics were compared to the study dogs as detailed below.

### Demographics and baseline clinical features

Signalment of dogs and baseline severity of OA and associated pain are detailed in Table 1. Briefly, among 42 study dogs, 23 were males, the remaining being females; mean age was 9 ± 3.2 years and mean weight 31 ± 10.5 kg. Mixed-breed and Labrador retriever were the most commonly represented dogs (*n* = 8, 19% each), followed by German shepherd (*n* = 7, 17%), Cane Corso and Golden retriever (*n* = 3, 7% each), with all the other breeds being represented by one or two dogs each. The number of involved joints ranged from 1 to 5 and pain was scored as severe on HCPI (range 18–39). Moreover, the vast majority of the study dogs (84%, *n* = 35) had pain on palpation scores corresponding to moderate-to-severe pain and similar results were observed for lameness (Table 1).

**Table 1:** Signalment of enrolled and study dogs

	**Enrolled dogs (*n* = 58)**	**Study dogs (*n* = 42)**
Sex	F 25 (Fs 21), M 33 (Mc 7)	F 19 (Fs 17), M 23 (Mc 5)
Age, years (mean ± SD; range)	8.9 ± 3.8; 12 mths – 16 yrs	9 ± 3.2; 13 mths – 14 yrs
Body weight, kg (mean ± SD)	32 ± 11.7	31 ± 10.5
Pain duration, months (mean ± SD)	22 ± 19.4	20.1 ± 17.8
No. of involved joints (mean; range)	2.4; 1–5	2.3; 1–5
Pain on HCPI (mean ± SD; range)	29 ± 4.76; 18–39	29 ± 4.77; 18–39
Pain on palpation	Moderate (*n* = 30, 52%) Severe (*n* = 18, 31%)	Moderate (*n* = 20, 48%) Severe (*n* = 15, 36%)
Lameness severity	Moderate (*n* = 27, 47%) Severe (*n* = 24, 41%)	Moderate (*n* = 16, 38%) Severe (*n* = 20, 48%)

*Abbreviations*: *F* Female, *Fs* Spayed female, *M* Male, *Mc* Castrated male, *mths* Months, *yrs* Years

The NSAID nonresponder group did not show any statistically significant difference from the responder group in any of the baseline features. At logistic regression, none of the analyzed variables resulted to be a strong predictor for the nonresponse to the full NSAID dose, with the exception of the number of OA affected joints, which however did not reach statistical significance [*P* = 0.0529, OR 1.66 (CI 95% 0.99–2.77)].

### Primary outcome

Based on Kaplan–Meier estimate of success rate, 90% of the study dogs achieved reduction of NSAID up to 25% of the original dose without pain worsening. Moreover, pain was considered under control (i.e., either decreased or unchanged compared to the previous timepoint, according to the owner’s assessment) up to 2 and 10 weeks after NSAID withdrawal in 77% and 75% of study dogs, respectively (Table 2). Interestingly, at the Cox proportional hazard model, none of the demographic nor clinical features at presentation significantly influenced the success rate (i.e., maintenance of pain relief).

**Table 2:** Success rate of the dietary administration of PGA-cur in relation to the NSAID dose reduction

Percentage of the original NSAID dose	75	50	25	0	0
Time from NSAID withdrawal (weeks)	-	-	-	2	10
Success rate (Kaplan–Meier estimate)	95%	93%	90%	77%	75%

The percentage of dogs whose pain was considered under control during the NSAID tapering phase and beyond was the primary outcome of the study and is here indicated (last line) in relation to meloxicam dose reduction

### Secondary outcomes

#### Time to pain worsening

**Fig. 2:**
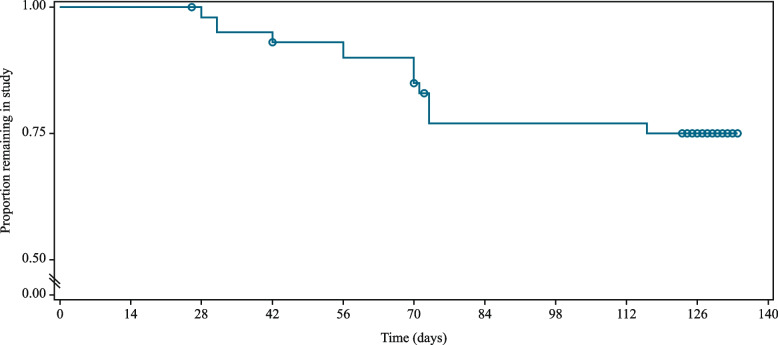
Survival plot, determined using Kaplan–Meier estimator, for time-to-pain worsening (days) for the study dogs. Circles indicate censored observations. The last group of circles refers to dogs that ended the study without worsening, with one circle representing more than one dog if censoring occurred on the same day

The mean time to pain worsening was 102.7 ± 4.3 days, with the value being largely underestimated due to the high number of dogs who reached the study end without worsening (*n* = 29 out of 42, 69%) (Fig. 2). Due to the same reason, the median time to pain increase, that is the length of time corresponding to the probability of 0.5, was not estimable, being unavoidably longer than the whole study duration (Fig. 2). Five dogs had the final visit (V3) performed 1, 2, 3, 4 and 5 days later than the scheduled timepoint.

#### Change in HCPI and CBPI scores

**Fig. 3:**
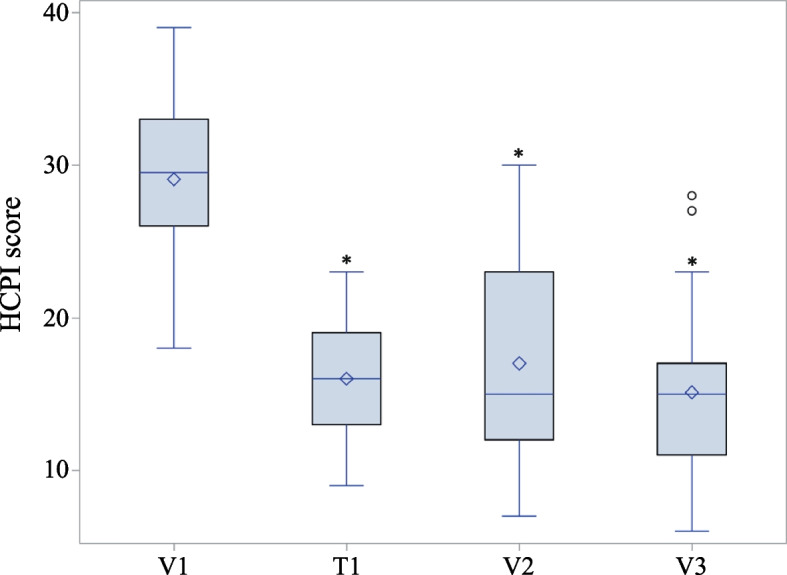
Box plot of pain severity on HCPI at the study timepoints. Diamond represents the mean; the line through the box, the median; circles are outliers falling outside that range. * *P* < 0.0001 *vs* V1. Abbreviations: V1, baseline visit; T1, first telephone interview; V2, intermediate visit; V3, final visit. See Fig. 4 for timeline and Methods for further details

The multimodal protocol under investigation resulted in a significant relief of chronic pain (*P* < 0.0001) as scored at HCPI (Fig. 3). In particular, the mean HCPI score decreased from baseline by 45% during the first two weeks (i.e., NSAID full dose). The mean decrease during the study period, i.e., V1-V3, was 13.9 ± 1.13 (- 48%), with a more sustained reduction between V1 and V2 (- 41%) and a further, albeit smaller, decrease from V2 to V3 (- 11%) (Table 3). Reduction of at least 50% from baseline HCPI score was achieved by 14 out of 29 dogs (48.3%).

**Table 3:** Decrease of pain score compared to V1 (i.e., baseline) as assessed by HCPI

	T1	V2	V3
*n*	42	35	29
Range	8 – 25	-3 – 23	1 – 26
Median	13	12	15
IQR	(9 to 15)	(8 to 18)	(9 to 18)
Mean	13.0 (-45%)	12.2 (-41%)	13.9 (-48%)
StdErr	0.62	1.14	1.13

*Abbreviations*: *IQR* Interquartile range, *T1* First telephone interview, *V2 and V3* Intermediate and final visit, respectively (see Fig. 4 for timeline)

During the study, the percentage of dogs with severe pain (HCPI ≥ 17) decreased from 100 to 41%. At V3, dogs with mild to moderate pain (12 ≤ HCPI ≤ 16) and those with uncertain pain (7 ≤ HCPI ≤ 11) or without pain (HCPI ≤ 6), accounted for 31% and 28% of the sample, respectively.

During the last ten weeks of the study (i.e., following NSAID withdrawal), no statistically significant change was observed in the total, severity and interference CBPI scores (Table 4). The mean total CBPI score at the beginning of this period was low (22% of the maximum possible score, Table 4) and did not change throughout the study. Similar findings were observed for PSS and PIS sub-scores, as detailed in Table 4. At the end of the combined treatment period (T4), quality of life as scored by the last question of CBPI was considered good-to-excellent in over 90% of the study dogs and did not significantly change throughout the study.

**Table 4:** Pain scores at different timepoints as assessed by CBPI

	Total pain score			PSS			PIS		
	T4	T5	V3	T4	T5	V3	T4	T5	V3
*n*	36	29	29	36	29	29	36	29	29
Range	0–49	8–37	0–72	0–3.8	1–3.8	0–7	0–5.7	0.5–4.2	0–7.3
Median	19	18	21	1.8	1.8	2.0	1.9	1.7	1.8
IQR	(16 to 26.5)	(15 to 26)	(7 to 33)	(1.8 to 2.6)	(1.8 to 2.3)	(0.8 to 3)	(1.5 to 3)	(1.3 to 2.8)	(0.7 to 3.3)
**Mean**	**21.8**	**20.7**	**22.4**	**2.1**	**2.0**	**2.2**	**2.3**	**2.1**	**2.3**
StdErr	1.6	1.5	3.5	0.1	0.1	0.3	0.2	0.2	0.4

Total pain score, pain severity and pain interference scores on CBPI as registered at the different timepoints of the study are detailed. No statistically significant changes were observed at any time

**Fig. 4:**
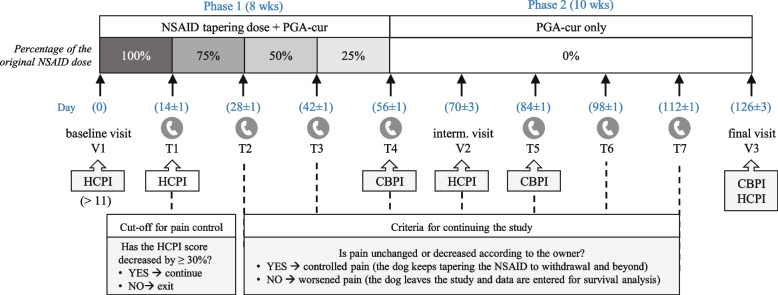
Timeline of the study. Timepoints of the two-phase study depicting the three clinical visits (V1, V2 and V3) and the seven telephone interviews (T1-T7) are shown. Timing for HCPI and CBPI administration to the owners are also indicated. The cut-off for pain control following the two-week administration of full dose meloxicam is shown (bottom left) and the criteria for continuing the study from T2 onward is also indicated (bottom right). Dotted lines represent timepoints in which the criteria for the maintenance of pain relief were checked

*Abbreviations*: *PIS* Pain Interference Score, *PSS* Pain Severity Score, *T4 and T5* Fourth and fifth telephone interviews, respectively, *V3* Final visit (see Fig. 4 for timeline)

#### Change in clinical scores

Both lameness and pain on palpation as assessed by the veterinary surgeon significantly changed to a less severe distribution over time (*P* < 0.0001) as summarized in Table 5.

**Table 5:** Distribution over time of the severity of lameness and pain on palpation

	*n* (%)		
	V1	V2	V3
**Lameness**			
0 - normal	0 (0.0%)	9 (25.7%)	10 (34.5%)
1 - slight/inconsistent	6 (14.3%)	18 (51.4%)	16 (55.2%)
2 - moderate	16 (38.1%)	7 (20.0%)	2 (6.9%)
3 - severe	20 (47.6%)	1 (2.9%)	1 (3.4%)
Comparison to V1		*P* < 0.0001	*P* < 0.0001
**Pain on palpation**			
0 - no signs	1 (2.4%)	14 (40.0%)	12 (41.4%)
1 - mild	6 (14.3%)	10 (28.6%)	13 (44.8%)
2 - moderate	20 (47.6%)	9 (25.7%)	4 (13.8%)
3 - severe	15 (35.7%)	2 (5.7%)	0 (0.0%)
Comparison to V1		*P* < 0.0001	*P* < 0.0001

The severity of lameness and pain on palpation was measured by the veterinary surgeon on two different 5-point scales at each clinical visit. No dog was scored 4 (extreme lameness / pain) at neither scale at any timepoint. The number and percentage of dogs for each severity class are here detailed

*Abbreviations*: *V1* Baseline visit, *V2* Intermediate visit, *V3* Final visit

#### Global assessment of efficacy perceived by the veterinary surgeon and owner

Once each of the study patient reached the study end, regardless of the reason why, the veterinary surgeon was asked to express an overall satisfaction on how OA pain was managed. No veterinary surgeon considered the treatment protocol as “insufficient” for managing OA pain. Veterinary surgeons rated the overall management as “good” for nearly half of the patients (*n* = 20, 49%), “excellent” for 29% (*n* = 12) and sufficient for 22% (*n* = 9). Datum was missing for one dog only.

All owners were also asked how their dogs’ pain was managed throughout the study. Nearly 80% answered pain was well-to-very well managed, 18% fairly managed and just one considered pain was poorly managed during the study. Data were missed for 3 dogs.

### Tolerability and safety

The multimodal protocol under investigation was overall safe and well tolerated. Only 8 dogs (14%) exhibited adverse events (AEs), with none being considered serious. All AEs were gastrointestinal in nature (i.e., 3 vomiting and 5 loose stools/diarrhoea) (Table 6). As detailed in the table, only two of the registered AEs resulted in dogs discontinuing the study. Finally, no AE manifested during the period of time when the dogs were administered the complementary feed only (i.e., from day 56 onward).

**Table 6:** Overall summary of adverse events (AEs) in chronological order (see Fig. 4 for timeline)

Onset (days in study)	AE description	Resulting in study exit	Purportedly associated with	Suspected causal relationship
2	vomiting	NO	PGA-cur	Probable
10	vomiting	YES	Meloxicam	Probable
10	loose stools	NO	PGA-cur	Probable
10	mild diarrhoea with mucus	NO	Meloxicam	Possible
14	chronic vomiting	NO	Meloxicam	Probable
15	loose stools	NO	PGA-cur	Probable
30	diarrhoea with mucus	NO	change in diet	Unlikely
42	diarrhoea	YES	Meloxicam	Probable

## Discussion

The results from the primary outcome showed that dietary supplementation with PGA-cur in dogs with moderate to severe OA pain allowed to progressively reduce the dose of meloxicam (up to discontinuation) without evidencing pain increase according to the owners. In particular, 90% of dogs could achieve a substantial dose reduction of meloxicam (up to reaching 25% of the full original dose) without resulting in pain increase based on owner assessment. Moreover, 77% and 75% of owners considered their dogs’ pain to be well controlled, two and ten weeks after meloxicam had been withdrawn, respectively. It should be acknowledged that a caregiver effect for owners could have influenced the success rate. Although no information is available on the size of this effect when dealing with pain assessment, this bias has been described for owners evaluating dog’s lameness and estimated to occur with a frequency of nearly 40% [33].

None of the demographic characteristics of the study dogs (e.g., gender, age, weight) nor any of their clinical features (e.g., disease duration, baseline HCPI, lameness and pain) was associated with pain worsening in response to meloxicam dose reduction. To our knowledge, only few studies have previously investigated the influence of dog’s features and disease severity on treatment response. In the study by Alves and colleagues [34], age showed to impact the duration of the effect of intra-articular interventions on the degree of lameness, stiffness and gait abnormalities in OA dogs [34]. On the contrary, no influence was shown on pain as assessed by the owner [34–36], which is in agreement with the present study. The fact that the variables of interest did not influence pain decrease means that the present study investigated the effect of the multimodal protocol, regardless of the particular features of the sample dogs.

Noteworthy, pain worsening was based on the owner’s assessment, with this more closely mirroring the clinical setting, where actually are the owners who perceive the effect of their dogs’ treatment.

These results are encouraging compared to a previous study in dogs progressively reducing the dose of meloxicam as a stand-alone treatment [19]. In particular, in the study by Wernham and colleagues the median time to pain increase was 84 days (twelve weeks) [19], while in the present study it was too long to be estimated due to the low proportion of dogs experiencing pain worsening before the study end (i.e., eighteen weeks). Although the lack of a control group should be kept in mind, the proportion of dogs considered to experience pain worsening during the study period (21%, Fig. 2) was smaller compared to a previous study, in which the dose reduction of meloxicam without any other intervention yielded higher worsening rate (i.e., 43%) [19]. Further factors, like body condition score, overall activity, age and severity at study entry could also have influenced the results and explain the different findings.

Interestingly, all dogs with pain worsening dropped out by the intermediate visit, suggesting that dogs whose pain was considered under control—based on owner’s perception—up to few weeks after meloxicam discontinuation would not experience flare-ups from that point onwards under the study supplement.

Before discussing further results, it is mandatory to keep in mind that while analysis performed on the primary outcome and time to pain worsening took into account data from the whole study sample (i.e., 42 dogs responding to meloxicam full dose administration) those performed on the other outcomes considered dogs whose pain did not worsen according to owners’ assessment. That being said, the treatment protocol here investigated allowed for a significant decrease of pain scored on HCPI. It is worth mentioning that enrolled dogs had severe pain at study entry, i.e., baseline HCPI scores ranging from 18 to 39 points, with the vast majority (86%) also presenting with moderate to severe lameness according to the veterinary surgeons. To the best of our knowledge, only few previous studies have used HCPI to evaluate treatment effect in OA dogs. The mean decrease of HCPI here observed (13.9 ± 1.13,—48%) far exceeded that reported in earlier studies, which evaluated oral as well as intra-articular treatment interventions, given to OA dogs as stand-alone or with a rescue NSAID [37–40]. Similarly, the mean decrease of HCPI observed in the present study was several folds higher than that reported in a previous trial on the effect of fish oil in dogs suffering from OA pain [41].

The results may be viewed as a confirmation of the utility and reliability of the multimodal approach to OA associated pain. OA is a chronic and complex disease of the whole joint, that inevitably progresses over time [42]. Previous experimental studies have shown that micronized PGA [26], and even more so co-micronized PGA-cur [31], is effective in counteracting multiple mechanisms of OA (i.e., degenerative, inflammatory and nerve sensitizing changes). It is possible that these effects synergized the well-known mechanisms of action of NSAIDs [43], similarly to what was found by adding amantadine to meloxicam in dogs with OA pain [44].

In line with previous findings on the ability of PGA, either alone or co-micronized with curcumin, to exert beneficial effects against inflammation and chronic pain [26, 27, 31, 32], here we found that PGA-cur not only maintained pain relief in dogs receiving a progressively reducing dose of meloxicam, but also resulted in a further (albeit smaller) decrease of pain severity after meloxicam withdrawal. This was evidenced by the decrease of HCPI, pain on palpation and lameness scores between the intermediate and the final timepoint. Unchanged pain scores on CBPI during the last ten weeks (i.e., after meloxicam withdrawal) is apparently in contrast with these findings. The ability of different metrology instruments to capture change of OA pain to variable degrees might account for the discrepancy [45]. On the other hand, the lack of statistically significant changes in pain scores on CBPI following meloxicam withdrawal might suggest PGA-cur prolonged meloxicam-induced pain relief during and after dose reduction. Further controlled trials are required to confirm or refute the hypothesis.

In the present study, 22% of dogs did not reach the minimum pain decrease during two-week meloxicam administration at full dose and were considered NSAID nonresponders. Although the proportion is higher than previously reported (i.e., up to 10%-12%) [46], overall the finding is consistent with the virtual lack of effect of meloxicam (0.2 mg/kg/day for three weeks) recently shown in dogs with bilateral hip OA [47]. As no significant differences were found in pain severity nor demographic features (e.g., age, body weight) between the NSAID nonresponders and study dogs, a different cause should be sought. Possibly, a neuropathic pain component not responding to the anti-inflammatory treatment might explain the finding. Alternatively, the arbitrary cut-off of 30% decrease in HCPI could account for the higher rate of response failure, although it should be noted that this percentage decrease corresponds to the average size of the effect in response to a 15-day stand-alone meloxicam treatment [19].

The overall incidence of AE in the whole study sample was low (14%) compared to previous studies [48]. They were all gastrointestinal in nature, none was serious and only two resulted in study exit (Table 6). This finding is particularly interesting in view of the commonly estimated 5%-10% of pets that have to discontinue NSAIDs because of AEs [46]. All AEs manifested during the NSAID administration (i.e., by the sixth week), with no AE being reported during PGA-cur given alone. This result agrees with the study by Asperio [32] and the overall safety profile of PGA and curcumin [20, 26, 49].

The present study has some limitations, with the first being the open nature of the design, which did not allow for comparison with a control group (i.e., dogs without PGA-Cur supplementation). However, it is worth mentioning that a previous study investigating meloxicam in tapering regimen as a stand-alone treatment for canine OA pain [19] found that dose reduction could be achieved in 57% of dogs, with the others (43%) dropping out due to pain worsening upon meloxicam dose reduction of 60%. Indeed, due to the inflammatory nature of OA pain, pain recurrence after NSAID dose reduction or discontinuation is a common clinic experience. Conversely, in this study 90% of dogs were still considered under control with respect to pain when administered 25% of the original full dose. Moreover, 77% and 75% of dogs did not show pain worsening up to 2 and 10 weeks after NSAID withdrawal, respectively. It may thus be hypothesized that the addition of PGA-cur to the meloxicam dose-tapering regimen contributed to pain control in three-quarters of the study dogs up to 10 weeks after NSAID withdrawal.

Another limitation of the study is that no objective measures (e.g., force plate analysis) were included and the evaluations only relied on subjective measures. However, it should be noted that gait analysis and metrology instruments are considered to quantify different aspects, and the latter is suggested when investigating the effect of the intervention on chronic pain rather than lameness [50].

With all these limitations in mind, the results of this study make dietary supplementation with PGA-cur an attractive adjunctive measure to NSAIDs in managing canine OA and associated chronic pain.

## Conclusions

The findings of the present study show that long-term dietary intervention with PGA-cur possibly contribute to maintain meloxicam-induced pain relief upon dose reduction in client-owned dogs with severe OA pain. This is interesting because of two main reasons. First, the long-term use of NSAIDs is debatable due to the risk of side effects, especially in frail animals (e.g., > 8 years of age; with pre-existing kidney, heart, and/or liver problems) [51–53]. Second, the beneficial effect of meloxicam is known to wean off soon after treatment discontinuation [47]. From a clinical perspective, maintaining the NSAID effect while reducing its intake is thus desirable in dogs suffering from OA pain. The potential NSAID sparing effect of PGA-cur warrants further clinical investigation.

## Methods

### Study aim and design

The aim of the present study was to investigate whether 18-week dietary administration of PGA-cur can maintain pain relief in dogs with OA-associated chronic pain, concurrently receiving a reference NSAID on a tapering regimen during the first 8 weeks of treatment, i.e., up to drug discontinuation.

The study was designed as a long-term, open label, multicenter study in dogs with OA chronic pain. Three assessment visits (V1, V2 and V3) were performed by veterinary surgeons at study entry, at two weeks (day 70 ± 3 days) and ten weeks (day 126 ± 3 days) after meloxicam withdrawal, respectively. Timing for the visits was based on a number of factors. In particular, the intermediate visit was set two weeks apart from meloxicam withdrawal in accordance with the aim of the study (i.e., to investigate if the dietary intervention under investigation was able to maintain meloxicam-induced pain relief after the analgesic effect of the latter had worn off). The timing was based on a previous study [19] as well as recommendations on NSAID washout periods [54] and reflected the “baseline” for PGA-cur. On the other hand, the last timepoint (i.e., the last visit at 126 days after the study entry) was chosen in order to have a longer observation period compared to a previous study on time to pain recurrence in dogs on meloxicam dose-reduction (i.e., 84 days) [19]. Seven telephone interviews (T1-T7) were administered every other week (± 1 day) by a single trained veterinary algologist from the Pain Therapy Service (Department of Veterinary Medicine, University of Perugia, G.d.R.). Further details of the study timeline are summarized in Fig. 4.

The study consisted in two parts. During the first phase of the study (eight week-duration), the NSAID was administered in a tapering dosage schedule along with an unchanged dose of the study supplement (see paragraph “ Products”). After the first two weeks of full dose NSAID administration, dogs who met or exceeded the arbitrarily set threshold of pain improvement (i.e., reduction of at least 30% in Helsinki chronic pain index, HCPI) continued the study and are referred to as “study dogs” throughout the paper. All the others were considered “NSAID nonresponders”, exited the study and did not enter the statistical analyses, in accordance with the study hypothesis. The 30% improvement level was based on clinical experience and previous studies on meloxicam clinical efficacy [13, 19].

Starting from the third week of the study, owners of the study dogs were instructed for progressively reducing the NSAID dose by 25% of the original dose every other week (see paragraph “ Products”), provided that they graded their dogs’ pain as unchanged or decreased during the respective telephone interviews by the Pain Therapy Service, similarly to [19]. Successfully managed dogs (i.e., those whose pain was unchanged or decreased at each control interview, according to the respective owner) discontinued the NSAID by the end of the eighth week (T4) and entered the second phase of the study (ten week-duration). During this phase, dogs were maintained on the supplement only and kept in the study as long as pain was “unchanged” or “decreased” according to the owner (please refer to Fig. 4 for visit and interview timing as well as criteria for continuing the study).

No anesthesia procedure was followed on animals throughout the study. Owners were informed that their dogs were allowed to leave the study at any time without consequences.

### Animals

Client-owned dogs, 12 months of age or older, of any breed or sex, with clinical and radiographical diagnosis of OA and chronic pain (i.e., lasting longer than 3 months and with HCPI score > 11) were included. The full list of inclusion and exclusion criteria is detailed in Table 7. The sample size was estimated to observe an improvement of 30% with respect to a null hypothesis of having an exit rate of 50% two weeks after meloxicam withdrawal, with α set to 0.05 and power to 0.8.

**Table 7:** Eligibility criteria for dogs to be enrolled in the study

**Inclusion criteria**
- Age ≥ 12 months
- Body weight ≥ 8 kg and ≤ 60 kg
- Clinical and radiological diagnosis of OA
- OA pain lasting ≥ 3 months
- Pain severity > 11 on HCPI
- At least two of the following HCPI items being described as “difficult” or “very difficult” by the owner (i.e., scored 3 or over): items 5, 7, 8 and 9, corresponding to the ability to trot (5), jump (e.g., in car, on sofa, 7,), lie down (8) and rise from a lying position (9)
- Pain on palpation or lameness being scored > 1 (each on a 0–4 scale) by the veterinary surgeon
- Stable home environment and lifestyle (including physical activity level) throughout the study
- Owner ensured that the same family member was to take the dog to each visit and answered the telephone questionnaire each time
**Exclusion criteria**
- Persistent or chronic pain due to any cause different from OA (e.g., cancer, neurological disorder)
- Any concurrent disorder interfering with dog’s locomotion, muscle function, physical activity or quality of life (e.g., hypothyroidism, hypo-/hyperadrenocorticism, electrolyte disturbance)
- Contraindications to the use of NSAIDs
- Pregnant or lactating dogs
- Long-acting steroids within 8 weeks before study entry
- Either oral/parenteral steroids or analgesics (e.g., gabapentin, amantadine, SSRIs) within 4 weeks before study entry
- NSAIDs the week before study entry
- Any concurrent treatment (including physical rehabilitation)
- Any surgery less than 12 months prior to the study

*SSRIs* Selective serotonin reuptake inhibitors

### Products

The study product was a complementary feed formulated in chewable tablets (Glupacur®, Innovet Italia, Milan, Italy). It contained a micronized composite (particle size range 0.6–10 microns) comprised of palmitoyl-glucosamine (266 mg per tablet) and *Curcuma longa* extract (133 mg per tablet) in a 2:1 ratio. The daily dose was based on the manufacturer’s instruction, ranging from one tablet (dogs 8–14.5 kg b.w.) up to three tablets (dogs 39.5 kg b.w. and over) and remained unchanged throughout the entire study.

During the first phase of the study, the complementary feed under investigation was administered along with meloxicam 1.5 mg/mL oral suspension (Metacam®, Boehringer Ingelheim Vetmedica GmbH, Ingelheim/Rhein, Germany), given daily by the owner, with food, at the following tapering regimen: weeks 1–2 (up to T1), 0.1 mg/kg/day (original dose); weeks 3–4 (from T1 to T2), 0.075 mg/day (75% of the original dose); weeks 5–6 (from T2 to T3), 0.05 mg/kg/day (50% of the original dose); weeks 7–8 (from T3 to T4), 0.025 mg/kg/day (25% of the original dose). No other products nor any physical rehabilitation procedures were allowed throughout the study.

### Measures

Two owner-based metrology instruments, i.e., the HCPI and CBPI, were administered to dog owners, either at clinical visits by the veterinary surgeon or during telephone interviews as detailed in Fig. 1. Two clinical metrology instruments were used in order to capture various dimensions of OA [8]. The same family member completed all the questionnaires throughout the study. Owners were blinded to their previous answers, in order to minimize bias.

The HCPI is an 11-item instrument, with 0–4 score of each item being summed to give an overall chronic pain score (ranging from 0 to 44) [55]. Dogs were categorized as having either severe (HCPI ≥ 17), mild to moderate (12 ≤ HCPI ≤ 16), or uncertain pain (7 ≤ HCPI ≤ 11) and were considered pain-free for HCPI ≤ 6, as previously described [39, 41, 56, 57]. The Italian translation of the original HCPI, currently under validation process, was administered to dog owners at each clinical visit (V1, V2 and V3). In order to verify whether the full-dose course of meloxicam relieved pain, HCPI was also administered during the first telephone interview (T1).

The CBPI is composed of two parts: the Pain Severity Score (PSS) and Pain Interference Score (PIS). PSS and PIS are the arithmetical mean of four and six items respectively, each item being scored on a 0–10 scale [58]. In addition, a stand-alone item is included at the end of the questionnaire to obtain the owner’s assessment of the dog’s quality of life (“poor”, “fair”, “good”, “very good”, excellent”). The recently validated Italian version of CBPI was used [59] and administered to dog owners at T4, T5 and V3, in order to monitor changes in pain severity after the NSAID withdrawal.

Moreover, at each telephone interview owners were asked whether pain was decreased, unchanged or increased with respect to the previous timepoint.

Although the owner is in a privileged position with respect to detecting changes in the dog’s behavior within the normal environment, a clinical evaluation of each dog by a trained veterinary surgeon was also included, in order to gain a more complete view of the dog’s pain and related functional limitations. To this end, lameness and pain on palpation were subjectively assessed by the veterinary surgeon on 5-point respective scales modified from previously published studies [39, 60, 61]. Assessment was performed at baseline (V1), intermediate (tenth week, V2) and final visit (eighteenth week, V3). Lameness was scored as 0 = stands, walks and trots normally; 1 = stands normally, slight lameness at walk or trot; 2 = stands normally, moderate lameness at walk or trot; 3 = stands normally, severe lameness at walk or trot; 4 = extreme lameness (not weight-bearing) at walk or trot [39]. Pain was scored as 0 = no sign of pain; 1 = mild pain (dog turns head in recognition); 2 = moderate pain (dog pulls limb away or wants to move away); 3 = severe pain (dog vocalizes or becomes aggressive); 4 = extreme pain (dog does not allow palpation) [39].

Global assessment of efficacy was performed at the study end by the veterinary surgeon and the owner through frequently used Likert-style scales [62]. The veterinarian was asked to grade his/her satisfaction on the overall patient management using a 4-point verbal rating scale (“poor”, “fair”, “good”, “excellent”), while the owner used an “emoji-based” 5-point smiley face scale (“very good”, “good”, “neither good nor bad”, “bad”, “very bad”) in order to judge how the joint pain of their dogs was managed.

### Outcomes

The primary outcome was the percentage of dogs in which pain improvement obtained with two week-NSAID treatment at the full dose (i.e., at least 30% decrease of the respective HCPI score compared to baseline) was maintained during the tapering phase and beyond. The above-described primary outcome is referred hereafter as success rate. The secondary outcomes were the followings: (i) time to pain worsening (i.e., the time in which pain did not increase despite dose reduction and subsequent withdrawal of the NSAID), (ii) change over time of HCPI and CBPI scores, (iii) different distribution over time of the severity of clinical scores (i.e., lameness and pain on palpation), (iv) global assessment of efficacy perceived by the owner and veterinary surgeon.

### Tolerability

Tolerability was assessed by monitoring adverse events (AEs) and animal withdrawals at any time during the study. An AE was defined as “any observation in animals, whether or not considered to be.

product-related, that is unfavorable and unintended and that occurs after any use of the study product(s)” [63]. All untoward effects that occurred during the study were recorded by the investigating veterinary surgeons in the case report form of each patient. Onset, severity (“not serious” and “serious”, i.e., fatal, life-threatening or resulting in persistent disability) and perceived causal relationship with the study intervention ( “probable”, “possible”, “unlikely” or “unclassifiable”) were recorded, in accordance with the ABON system, as recommended by the EMEA Committee for Veterinary Medicinal Products [63, 64].

### Data analysis

All data were analyzed using SAS v9.2 (SAS Institute, Cary; NC, USA). Descriptive statistics were used to describe demographic characteristics of the enrolled subjects (mean ± standard deviation, SD). When analyses on means were carried out (for primary and secondary outcomes) mean ± standard error (SE) was used. To test for homogeneity between NSAID responder and nonresponder group, Fisher’s exact test and T-test with unequal variance were used for nominal and continuous variables, respectively. Gender, age, body weight, disease duration, number of joints involved, OA origin (i.e., primary vs secondary OA), as well as baseline scores of HCPI, pain and lameness were the variables considered. In order to verify if any variable could predict unresponsiveness to the used NSAID, the estimation of odds ratio (OR) for individual variables (i.e., the association between each variable and failure to respond to full-dose NSAID) was assessed through logistic regression. The time to event (i.e., the time to pain worsening) was analyzed using the Kaplan–Meier survival analysis. Dogs that exited the study due to pain worsening were classified as "events", while all other reasons were considered "censorship". The Kaplan–Meier estimate of success rate was reported. To further explore for predictors of dropout, a stepwise procedure was performed with Cox proportional hazard model containing baseline demographic and clinical features of the study dogs. Changes in HCPI and CBPI scores over time were analyzed using the generalized linear mixed model (GLMM). The fixed effect in the model was time, the random effect was the animal. The veterinary surgeon was included as a covariate. Fisher exact test was used to analyze the distribution over time of pain and lameness severity scores as assessed by the veterinary surgeon. The level of significance was set at *P* < 0.05. Exact *P* values are reported, except less than 1 out of 10,000 (reported as *P* < 0.0001), with 0.0001 being the lower limit for the statistical program.

## Data Availability

The datasets used and analysed during the current study are available from the corresponding author on reasonable request.
